# Multi‐Strain Probiotic and Common Infections in Early Childhood Education Settings: A Randomised Controlled Trial

**DOI:** 10.1111/jpc.70295

**Published:** 2026-01-23

**Authors:** Hafiz Haris Ahmad, Blake Peck, Daniel Terry

**Affiliations:** ^1^ Institute of Health and Wellbeing, Federation University Ballarat Australia; ^2^ Department of Radiation Oncology Peter MacCallum Cancer Centre Melbourne Australia; ^3^ School of Nursing and Midwifery, University of Southern Queensland Toowoomba Australia; ^4^ Centre for Health Research, University of Southern Queensland Toowoomba Australia

**Keywords:** children, clinical trial, infections, probiotics

## Abstract

**Objective:**

This randomised controlled trial aimed to evaluate the effect of a multi‐strain probiotic on the incidence of common infections among children in early childhood education (ECE) settings.

**Methods:**

Prospective, randomised, double‐blind, placebo‐controlled trial was conducted using a multi‐strain (mixture of 5 strains) probiotic at a daily dose of 10 billion active fluorescent units. Participating children were randomly assigned to either the intervention or control group. The supplementation period lasted 24 weeks, during which weekly questionnaires were administered to track the incidence of infections.

**Results:**

A total of 118 children were enrolled in the trial. An intention‐to‐treat analysis revealed a 62% reduction in the incidence of gastrointestinal tract infections (GITIs) (incidence rate ratio: 1.62, *p* = 0.055) between the placebo and probiotic groups in the last 16 weeks of the study. Notably, it took up to 8 weeks for probiotics to exhibit a significant protective effect. However, probiotic supplementation had no impact on respiratory tract infections (RTIs). Additionally, probiotic use led to an estimated cost saving of AU$4748 in relation to reducing GITIs for 16 weeks after the protective effect was achieved.

**Conclusion:**

Multi‐strain probiotic has the potential to reduce the risk of GITIs among children in ECE settings, though no beneficial effect was observed on RTIs despite recording over 450 infections. Larger, multi‐arm trials are recommended to further investigate this area.

**Trial Registration:** ClinicalTrials.gov identifier: ACTRN12622000153718.

## Abstract (highlights)

**Summary**

- What is already known on this topic
  ○ Early childhood education (ECE) attendance is associated with an increased risk of infections.
  ○ Probiotics have the potential to influence the immune system.
- What this paper adds
  ○ In this study, multi‐strain probiotic reduced gastrointestinal tract infections (GITIs) among children in ECE settings. This is the first study to suggest that probiotics may require approximately 2 months to take effect.
  ○ Probiotic use saved AU$4748 in infection‐related costs within 4 months.

## 1 Introduction

Early childhood education (ECE) settings are vital to society, providing essential support to families by fostering the learning and development of young children, often while parents are at work [1]. However, both Australian and international research indicate that attendance at ECE settings is associated with an increased risk of infections [2, 3, 4, 5, 6]. Moreover, this issue impacts more than just children and negatively affects parents, employers and the healthcare sector [7, 8].

Probiotics are live microorganisms that, when consumed in adequate quantities, offer health benefits to the host as they can aid the colonisation of beneficial bacteria [9]. The use of probiotics has garnered significant attention from researchers due to their potential impact on immune function [10]. However, previous studies have produced mixed results [11, 12, 13, 14], and there is a lack of research on the minimum time required for probiotics to demonstrate a significant prophylactic effect against infections. Additionally, no randomised controlled trial has been conducted in Australia to examine the effects of multi‐strain probiotics in ECE settings.

A randomised controlled trial was conducted to evaluate the impact of probiotic (containing a mixture of five strains) use on the incidence of common infections including GITIs and RTIs among children in ECE settings. A secondary aim was to determine the minimum duration required for probiotic to become effective, assess its role in reducing the severity of infections, healthcare utilisation, and evaluate the economic impact of probiotic supplementation.

## 2 Methods

### 2.1 Study Design, Intervention and Setting

The trial was a prospective, randomised, double‐blind, placebo‐controlled study with the randomisation ratio of 1:1. The trial utilised a Therapeutic Goods Administration (TGA) Australia approved multi‐strain probiotic (Biome Daily Kids Probiotic; approval no: 317898) at a dose of 10 billion active fluorescent units (AFU) per day (one sachet per day). The probiotic contained five strains: *Lactobacillus plantarum* 6595 (ATCC 53103) (3 billion CFU), *Lactobacillus rhamnosus* GG (ATCC 53103) (2 billion AFU), *Bifidobacterium animalis* subsp. *lactis* BS01 (LMG P‐21384) (2 billion AFU), *Lactobacillus casei* LC03 (DSM 27537) (1 billion AFU), and *Bifidobacterium breve* BR03 (DSM 16604) (2 billion AFU). The placebo was packaged identically to the probiotic and contained the same carrier formulation but without the active bacterial strains. The appearance and taste of both the probiotic and placebo were identical, eliminating any possibility of distinguishing between the two study products. Similarly, none of the research personnel or participants were aware of the group allocations.

The study targeted children aged 2–5 years attending ECE settings in Victoria, Australia. Recruitment was conducted through childcare centres, kindergartens and social media.

### 2.2 Inclusion Criteria

- Children aged 2 years of age and older, as the study product is suitable for this age group.
- Attendance at an ECE for 2 days or more a week.
- If more than one child per family was recruited, both were assigned to the same study group (Group A or Group B) to prevent product mixing at home.

### 2.3 Exclusion Criteria

- Children older than 70 months at the time of enrolment.
- Children attending ECE settings for fewer than 2 days a week.
- Participants residing outside Victoria.
- Children currently receiving probiotic and/or prebiotic products whose parents were unwilling to discontinue these products 2 months prior to and during the study.
- Children receiving any medication that could interact with the probiotic supplement or affect the immune system or cognitive function.
- Children with severe chronic illnesses, including but not limited to neoplasm, immune deficiency, and chronic diarrhoea.

### 2.4 Ethics Statement

The study was approved by the Federation University Human Research Ethics Committee (no: A21‐163) and Victorian Department of Education (approval number: 2022_004555). The study was conducted in accordance with the principles of the Helsinki Declaration. It was prospectively registered with the Australian New Zealand Clinical Trials Registry (no: ACTRN12622000153718). All parents and guardians received study information in lay terms, and informed consent was obtained prior to the commencement of study procedures.

#### 2.4.1 Primary Outcomes

The primary outcomes of the study were the incidence of common infections, such as GITIs and RTIs, and absenteeism from ECE settings due to illness.

#### 2.4.2 Secondary Outcomes

Secondary outcomes included the duration of illness, incidence of fever, medication use, antibiotic use, doctor visits and cost of product.

### 2.5 Study Procedure

The study was conducted over 1 year (September 2022–October 2023), with the initial 5–6 months allocated for recruitment and baseline data collection. The intervention consisted of a 6‐month supplementation period (defined as 28‐day months) and a taper‐down month. From week 23, parents were instructed to reduce the daily dose (probiotic or placebo) to 4 doses in the first week, followed by 3, 2, and 1 dose per week over the next 3 weeks. The tapering‐down schedule was introduced to minimise the risk of any potential withdrawal symptoms associated with the abrupt cessation of daily probiotic intake. Similarly, supplementation began and ended simultaneously for all participants, running from March to August 2023, coinciding with the Australian winter when infection rates are typically high [15].

Following eligibility assessment by healthcare‐trained researchers, participants were randomly assigned to two groups using SPSS (version 28.0). Children were enrolled and randomised based on household structure. A single child from a household was randomised independently, while sibling pairs were randomised together to the same group to account for shared environmental factors and reduce confounding.

The study product was shipped to parents in two batches with detailed administration instructions. Parents completed an online weekly questionnaire (every Friday) to record new or ongoing infections and related symptoms. They reported missed sachets and infection symptoms including stool frequency, appetite loss, stomach pain, vomiting, runny nose and cough. RTI was defined as cold or flu or symptoms such as cough, sore throat or nasal congestion (with or without fever). GITI was defined as gastrointestinal symptoms such as diarrhoea, vomiting, appetite loss or stomach pain (with or without fever). An open‐ended section allowed reporting of other symptoms, later categorised as RTI, GITI or other infections based on their nature.

Additional weekly questions assessed disease severity, including duration, fever, doctor visits, medication or antibiotic use and hospitalisation. Parents were contacted every Friday afternoon and asked to respond by Sunday. A reminder email was sent each Sunday morning. This process continued without interruption throughout the supplementation period. Families were not financially compensated for participation.

### 2.6 Sample Size and Power Calculations

Based on previous studies [12, 13], probiotics were estimated to reduce the incidence of infectious diseases in childcare centres by 20%. Given that children in Australian childcare centres typically experience an average of 4 to 4.5 episodes of infectious diseases, a sample size of 106 was calculated using a 20% difference, 80% power and an alpha level of 0.05. Accounting for a 15% dropout rate, the final sample size was adjusted to approximately 120, with 60 children in each group. It should be noted that the referenced studies used different probiotic preparations, and the data were used solely as a reference for sample size estimation.

### 2.7 Statistical Analyses

Descriptive statistics were used to summarise gender, age, weight, household income, breastfeeding duration, and total infections in both groups. Differences were analysed using *t*‐tests and *Z*‐tests. Incidence rate ratios (IRRs) with 95% confidence intervals (CIs) and *p*‐values were calculated for infection incidence and associated factors (fever, doctor visits, medication and antibiotic use). Poisson and negative binomial regression were used to calculate IRRs, CIs and *p*‐values.

To estimate when the probiotic took effect, IRRs were calculated for each infection type over the full 24 weeks and the final 16 weeks. Infections showing significant reduction during either period were further assessed over the final 12 weeks to rule out one‐off occurrences and support a consistent downward trend.

While shortening the period may reduce events and compromise statistical significance, a consistent decline may still suggest clinical relevance and support the estimated onset of probiotic action [16, 17]. Other outcomes were analysed over the final 16 weeks, as statistical significance was observed only for GITI at that point.

All tests used a 5% significance level. Analyses were performed in SPSS Statistics for Windows, version 28.0 (IBM Corp., Armonk, NY, USA) on an intention‐to‐treat basis, including all randomised participants. Those who withdrew or stopped completing weekly questionnaires were considered lost to follow‐up. Each study month was defined as 28 days (4 weeks).

## 3 Results

### 3.1 Baseline Characteristics

**FIGURE 1:**
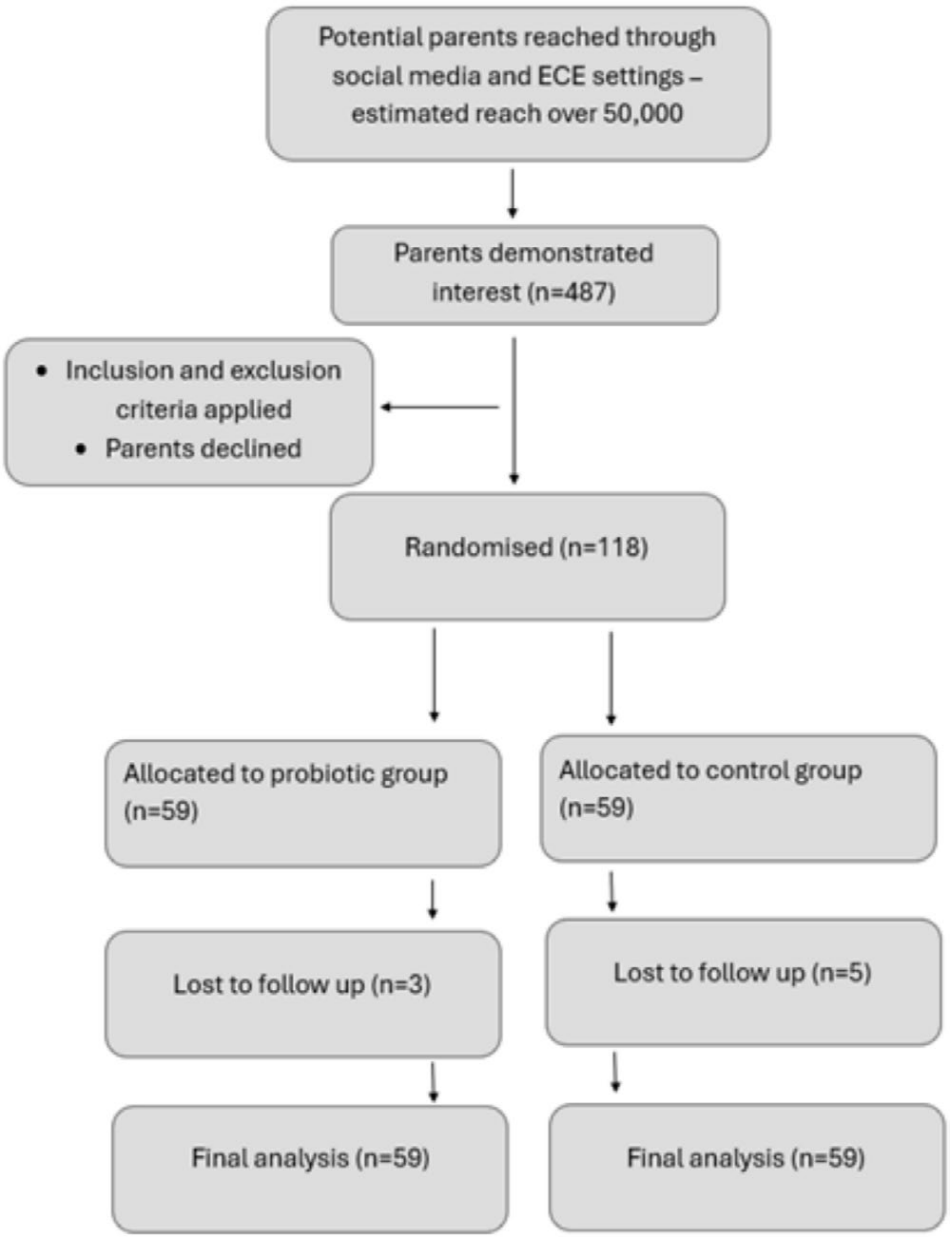
Flow diagram of participant recruitment, allocation, follow‐up and analysis in the trial.

A total of 118 children were enrolled in the trial, with 59 randomly assigned to the probiotic group and 59 to the control group (Figure 1). The study achieved ~95% retention, ~90% weekly survey completion, and a ~7% average missed dose rate. Baseline characteristics showed no differences between groups in gender distribution, household income, breastfeeding duration or average daily intake of yogurt (Table 1). Both groups had 39% (*n* = 23) male children, and the average weight was 16 kg in the control group and 15.9 kg in the probiotic group at baseline. The average age was 45 months in the control group and 42 months in the probiotic group. Average weekly income ranged from $1920 to $2399, and breastfeeding duration averaged 12–15 months in both groups. The groups showed minor difference in average daily consumption of fruits, vegetables, dairy, protein and cereal and grain intake (Table 1).

**TABLE 1:** Baseline characteristics of the study participants.

Variable name	Placebo (*n* = 59)	Probiotic (*n* = 59)
Male	23 (39%)	23 (39%)
Female	36 (61%)	36 (61%)
Weight (mean)	16.0 kg	15.9 kg
Age (mean)	45 months	42 months
Average weekly household income	$1920–$2399	$1920–$2399
Average breast‐feeding time	12–15 months	12–15 months
Average fruit consumption (per day)	2.5 serves	2.3 serves
Average vegetable consumption (per day)	1.9 serves	1.7 serves
Average yogurt consumption (per day)	0.6 serve	0.6 serve
Average dairy consumption (per day)	1.6 serves	1.5 serves
Average protein consumption (per day)	1.5 serves	1.4 serves
Average cereal and grain consumption (per day)	2.4 serves	2.6 serves

### 3.2 Monthly Infection Incidence, and Cumulative Incidence at 16 and 24 Weeks

**FIGURE 2:**
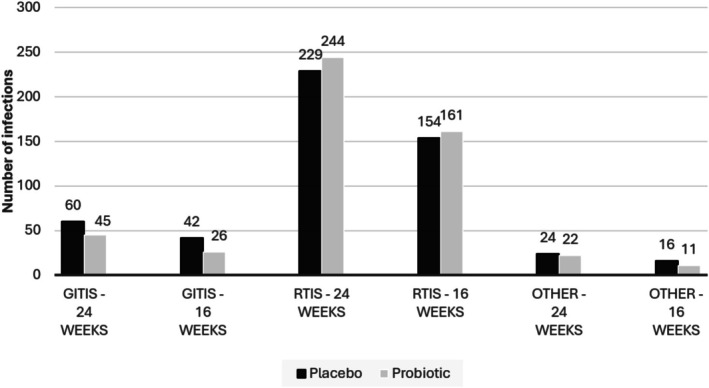
24‐weeks and 16‐weeks incidence of gastrointestinal tract infections (GITIs), respiratory tract infections (RTIs) and other infections in probiotic and placebo groups.

In the second month of supplementation, the incidence of infections was significantly higher in the probiotic group than the placebo group (Table 2). In the third month, the placebo group had the highest infection incidence, while the probiotic group's rate remained significantly lower (*p* = 0.008). Similarly, a marked difference was observed in total GITI incidence between the groups at both 24 (60 infections in placebo and 45 in probiotic) and 16 weeks (42 infections in placebo and 26 in probiotic) and the difference was more pronounced at 16 weeks (Figure 2). A similar pattern was observed for other infections at 16 weeks. However, RTI incidence remained similar between groups at both time points.

**TABLE 2:** Average monthly incidence (per child) of all infections in intervention and placebo group.

Months		Mean	Standard deviation (SD)	*T*‐test; *p*
1st month	Placebo	1.07	0.640	0.175
	Intervention	0.95	0.729	
2nd month	Placebo	0.58	0.563	**0.044** *
	Intervention	0.78	0.805	
3rd month	Placebo	1.34	1.060	**0.008** *
	Intervention	0.90	0.885	
4th month	Placebo	0.90	0.781	0.377
	Intervention	0.95	0.972	
5th month	Placebo	0.66	0.801	0.242
	Intervention	0.76	0.773	
6th month	Placebo	0.78	0.696	0.113
	Intervention	0.61	0.810	

* Bold indicates *p* ≤ 0.05.

### 3.3 Incidence Rate Ratios for Infections and Other Outcomes

The IRRs for GITIs, RTIs, other infections and healthcare utilisation outcomes over 12, 16 and 24 weeks are summarised in Table 3.

**TABLE 3:** Incidence rate ratios (IRRs) for infections and healthcare utilisation outcomes comparing intervention and placebo groups.

Outcome	Duration	IRR intervention (*n* = 59) placebo (*n* = 59)	95% CI	*p*
GITI	24 weeks	1.36	0.92–1.99	*p* = 0.122
GITI	16 weeks	1.62	0.99–2.64	***p* = 0.055** *
GITI	12 weeks	1.68	0.96–2.97	*p* = 0.072
RTI	24 weeks	0.94	0.78–1.12	*p* = 0.490
RTI	16 weeks	0.96	0.77–1.19	*p* = 0.693
Other	24 weeks	1.09	0.61–1.97	*p* = 0.772
Other	16 weeks	1.45	0.68–3.13	*p* = 0.339
Fever	16 weeks	1.23	0.79–1.92	*p* = 0.366
Doctor's visit	16 weeks	1.07	0.75–1.52	*p* = 0.717
Medication use	16 weeks	0.97	0.77–1.21	*p* = 0.773
Antibiotic use	16 weeks	1.50	0.80–2.82	*p* = 0.211

*Note*: IRR values greater than 1 indicate higher incidence in the Placebo group compared with the Intervention group.

Abbreviations: 95% CI, 95% confidence interval; IRR, incidence rate ratios.

* Bold indicates *p* ≤ 0.05.

For GITIs, the intervention group experienced a consistently lower incidence compared to the placebo group. For the last 16 weeks, the IRR was 1.62 (95% CI: 0.99–2.64; *p* = 0.055), indicating a 62% reduction in GITI risk in the intervention group. Similar trends were also observed at 12 weeks (IRR = 1.68; 95% CI: 0.96–2.97; *p* = 0.072), although these were not statistically significant (Table 3).

For RTIs, no significant differences were observed between groups at either 16 weeks (IRR = 0.96; 95% CI: 0.77–1.19; *p* = 0.693) or 24 weeks (IRR = 0.94; 95% CI: 0.78–1.12; *p* = 0.490). The incidence of other infections remained comparable between groups at 24 weeks. However, at week 16, the IRR was non‐significantly reduced in the intervention group (IRR = 1.45; 95% CI: 0.68–3.13; *p* = 0.339).

Lastly, no statistically significant differences were observed for other outcomes at 16 weeks: fever, doctor's visits, medication use or antibiotic use (Table 3). However, more children in the placebo group were significantly (*p* < 0.05) more likely to miss school compared to the treatment group (Table 4).

**TABLE 4:** Mean duration of illness and education missed in the intervention and placebo groups.

Events	Placebo (*n* = 59)	Treatment (*n* = 59)	*t*‐test or *Z*‐test
Average illness duration (Days) and SD	17.58 (12.62)	18.22 (14.36)	*t*‐test, *p* = 0.398
Average days education missed and SD	3.81 (4.29)	3.20 (4.51)	*t*‐test, *p* = 0.227
Number of children who missed 0 education days in the last 16 weeks of the study	10	19	*Z*‐test, *p* > 0.05
Number of children who missed 0 education days in 24 weeks of the study	4	13	***Z*‐test, *p* < 0.05** *

Abbreviation: SD, standard deviation.

* Bold indicates *p* ≤ 0.05.

### 3.4 Cost Analysis and Adverse Effects

The study observed a reduction of 16 GITIs in the last 4 months (16 weeks) of the study period (Figure 2 and Table 3 demonstrating reduction in incidence). Based on the cost of a single influenza‐like infection, AU$813 in 2023 [7, 8], the total cost savings were estimated to be AU$13 008 among the study cohort. The probiotics used retail for about AU$35 per box (30 sachets/1 month supply) [18], costing AU$8260 for 59 children over 4 months (16 weeks), resulting in a net saving of AU$4748 over 4 months. The study did not include a formal adverse‐effect analysis; however, no significant adverse effects were reported with probiotic use. The only reported issue was a transient change in stool consistency (soft stools) following initiation of the probiotic in the intervention arm, which resolved within a few days with continued use.

## 4 Discussion

This is the first randomised controlled trial in Australia to investigate the impact of a multi‐strain probiotic on the incidence of common infections among children in ECE settings and to assess the minimum probiotic action time globally. The study maintained strong retention and data completeness, with a low average rate of missed doses, which strengthens confidence in the results.

The study demonstrated that it may take approximately 2 months (8 weeks) for the combination of five probiotics used in this study to establish a protective effect. This estimate is based on the difference in average infection incidence in the third month (Table 2: 1.34 in placebo vs. 0.90 in probiotic group, *p* = 0.008) and the 62% reduction in IRR for GITIs (Table 3: IRR = 1.62, 95% CI: 0.99–2.64; *p* = 0.055). GITIs showed a non‐significant reduction over the full 24 weeks, then a 62% difference during the last 16 weeks, and a stronger (non‐significant) influence in the final 12 weeks, likely due to low counts. Considering the complex mechanism of probiotic action, this two‐month period is a reasonable timeframe, as the immune system requires time to be modulated [19]. There are no comparable studies directly supporting this timeline. However, a study by Hojsak et al. [20] in hospitals showed no effect of *Bifidobacterium animalis* subsp. *lactis* in preventing nosocomial infections, including GITIs, in children over 1 year (short‐term probiotic effect assessed) [20].

Similarly, the incidence rate of GITIs was around 36% higher in the placebo group compared to probiotic for the total duration of study (24 weeks), which increased to 62% for the last 16 weeks and 68% for the last 12 weeks of the study, which also shows substantial clinical significance of probiotic supplementation to manage GITIs in this cohort [16, 17]. These findings are consistent with past studies in similar areas, such as the meta‐analysis by Ahmad et al. [21], which found that probiotics (excluding *B. animalis* subsp. *lactis* BB‐12 strain) may reduce the risk of GITIs by 26%. The stronger effect observed in our study is likely due to the combination of five different probiotic strains used.

During the six‐month study period, over 450 RTI incidences were recorded across both groups. Despite this high disease burden, the trial did not show a significant reduction in RTI incidence. This aligns with the 2018 meta‐analysis by Laursen and Hojsak [14], which also found no effect of probiotic supplementation on RTI incidence. Our findings suggest that the five‐strain probiotic combination was insufficient to modulate the immune system against such a high incidence of diseases, particularly those affecting areas distant from the probiotics' local action site (GIT). Constant exposure to respiratory pathogens likely limited the probiotics' ability to influence the immune system via mechanisms such as mucosal wall strengthening and modulation of T‐lymphocytes and phagocytes [9, 22, 23].

The probiotic also showed limited effect on infection severity. No significant reduction was observed in doctor visits, medication or antibiotic use, fever incidence, or illness duration. This may be due to the high RTI‐to‐GITI ratio (~5:1). Since these health outcome measures were shared between RTI and GITI, and RTIs were five times more common but not influenced by probiotic use, no overall difference was detected. However, a statistically significant difference was seen in the number of children who missed no educational days during the study, with 13 in the probiotic group versus 4 in the placebo group. This correlates with the observed reduction in GITI risk in the probiotic group.

In terms of cost analysis, the probiotic use resulted in a net saving of AU$4748 over 4 months. However, it is important to recognise that these estimates are likely conservative, and actual cost savings may be substantially higher. A recent study conducted in the ECE settings in the United States estimated that the cost of a single episode of acute GITI exceeds AU$1500 (approximately AU$2300 based on the exchange rate as of 4th May 2025) per child [24]. Thus, the use of probiotics was considered economically feasible. Similarly, in terms of safety, the study did not identify any significant adverse events with probiotic use.

Several limitations are acknowledged. Although data were collected weekly, recall bias could have occurred. However, due to the study's randomised design, this bias would not have affected the conclusions. It is also possible that not all probiotic sachets were fully consumed, or some product was lost during mixing with food, which could have influenced the findings. Regarding GITI incidence and statistical significance, the study was limited by small sample size. Another limitation is the sample type: children were from a developed nation, which may reduce generalisability to developing countries. Despite these limitations, the findings hold noteworthy significance and the following recommendations are offered.

Clinicians may consider probiotics for young children experiencing a high burden of illness, particularly gastrointestinal infections. A combination of strains with a daily dose of 10B AFU is more likely to yield positive outcomes. Probiotics may also be consumed continuously, including during infections. Effects are likely to emerge after 8 weeks of use, and decisions about continuation can be made after this period. Future studies should involve larger probiotic trials with over 500 children in ECE settings over longer durations (a year or more) and include comparisons of multiple‐strain combinations, single strains, and a control arm.

## 5 Conclusion

The study concludes that the combination of five probiotic strains used may reduce the incidence of GITIs among children attending ECE settings. This suggests that probiotic supplementation may require 8 weeks to demonstrate a protective effect. However, despite recording over 450 RTI infections, this study did not find any positive influence of probiotic supplementation on the incidence of RTIs. Overall, probiotics were found to be safe and cost‐effective for reducing the risk of GITIs in this population.

## Funding

The study is supported by the Australian Government Research Training Program (RTP) place. The probiotics and placebo were provided in‐kind by Biome Australia Limited.

## Conflicts of Interest

The authors declare no conflicts of interest.

## Data Availability

The authors have nothing to report.
